# ADHD and Bipolar Disorder in Adulthood: Clinical and Treatment Implications

**DOI:** 10.3390/medicina57050466

**Published:** 2021-05-10

**Authors:** Virginio Salvi, Enrico Ribuoli, Michele Servasi, Laura Orsolini, Umberto Volpe

**Affiliations:** 1 Unit of Clinical Psychiatry, Ospedali Riuniti, 60100 Ancona, Italy; michele.servasi@ospedaliriuniti.marche.it (M.S.); laura.orsolini@ospedaliriuniti.marche.it (L.O.); u.volpe@univpm.it (U.V.); 2 Department of Clinical Neurosciences/DIMSC, Università Politecnica delle Marche, 60121 Ancona, Italy; enricoribuoli@gmail.com

**Keywords:** ADHD, bipolar disorder, comorbidity, stimulants, treatment-induced mania

## Abstract

Attention deficit hyperactivity disorder (ADHD) is a condition that usually has its onset in childhood. Although the disorder persists into adulthood in half of cases, adult ADHD is often not recognized due to different psychopathological characteristics, quite often overlapping with other diagnoses such as mood, anxiety and personality disorders. This is especially true for bipolar disorder (BD), which shares several symptoms with adult ADHD. Moreover, besides an overlapping clinical presentation, BD is often co-occurring in adults with ADHD, with comorbidity figures as high as 20%. This review will focus on the comorbidity between ADHD and BD by exploring the magnitude of the phenomenon and evaluating the clinical and functional characteristics associated with ADHD–BD comorbidity in adults. Finally, the review will address the implications of pharmacologically treating the ADHD–BD comorbidity, providing suggestions in how to treat these complex patients and addressing the issue of treatment-induced manic switch with the use of stimulants and other medications for ADHD.

## 1. Introduction

Attention deficit/hyperactivity disorder (ADHD) is a typical childhood-onset disorder characterized by a deficit of attention and motor hyperactivity leading to significant impairment in academic/occupational, familiar and social functioning [1]. In most cases, adult ADHD has a heterogeneous clinical presentation besides the hyperactivity and inattention described in the pediatric population, which includes a wider spectrum of emotional dysregulation and functional impairment [2].

ADHD has an estimated childhood prevalence of 4–7% [3], while its prevalence in adulthood is around 2.5% [4]. A systematic review of prevalence studies suggests that childhood ADHD persists into adulthood in 15–60% of cases [5]. Studies in children and adolescents showed that the disorder is at least 3-times more prevalent in males, while the male/female ratio tends to decrease to 2:1 in adults [6,7]. The observed gender difference may be explained with the higher frequency of hyperactivity and behavioral problems in boys, which make them more likely to be referred to the clinical specialist. Conversely, girls with ADHD show more attentional symptoms and fewer hyperactive/impulsive symptoms, which, possibly alongside greater coping skills, reduce the likelihood of referral [8]. Gender differences also appear to influence the prevalence of comorbid disorders. In particular, women suffering from ADHD are more prone to develop comorbidities with depressive and eating disorders, while males more likely suffer from comorbid substance use disorders [9,10,11,12].

In adults with ADHD the clinical expression of core symptoms is different compared with that observed in children and adolescents [13]. Adults often complain of inner restlessness, express excessive talkativeness and the need to move even in situations where one is expected to stand still. They may be impulsive and impatient, may “act without thinking” and sometimes get into trouble because of this feature. On the functional standpoint they may be unable to hold a job or maintain personal relationships. Other complaints, such as feeling bored, being unable to make decisions, procrastinating and being disorganized and distracted are often an expression of inattention in adulthood [13]. This diversity in clinical presentation, along with the general dearth of general psychiatrists with ADHD expertise, possibly contributes to the diagnostic gap between children and adults.

Another issue that further complicates the diagnostic process in ADHD adults is the high frequency of comorbid disorders, particularly mood, anxiety, personality and substance use disorders. Among these, bipolar disorders (BD) have overlapping symptomatology that may be frequently mistaken for ADHD, thus substantially contributing to its underdiagnosis and subsequent undertreatment.

Therefore, due to the many similarities between adult ADHD and BD and the need for clinical guidance in the diagnosis and management of both conditions, we aimed at reviewing the published literature on adult ADHD comorbidity with an in-depth analysis of BD comorbidity. Lastly, we discuss the implications and issues that arise when treating adults with co-occurring ADHD and BD.

## 2. ADHD and Comorbid Psychiatric Disorders

Several studies have found a high prevalence of comorbid psychiatric disorders in adult ADHD [9,10,11]. The high comorbidity rates may overshadow ADHD presentation, thus hindering the recognition and diagnosis of ADHD in adults. This is acknowledged as one of the main reasons for the observed under-recognition and undertreatment in the adult population [14].

The most frequently represented psychiatric comorbidities are mood and anxiety disorders, substance use disorders (SUD) and personality disorders, with rates for at least one comorbid psychiatric disorder ranging from 57 to 92% [9,11,15,16]. Other authors, adopting a dimensional approach to diagnose ADHD and comorbid disorders, found rates as high as 80% [17].

Mood disorders are especially common in patients with adult ADHD. A large epidemiological study conducted in 20 countries found a 12-month prevalence of major depression in the 15% of adult subjects diagnosed with ADHD [18]. Clinical studies reported even higher rates of lifetime major depression in adults with ADHD, ranging from 45 to 55% [9,17,19] The presence of a depressive disorder reduces the quality of life and overall functioning of patients with ADHD, further increasing the burden of disease [19,20]. In some cases depression arises as a result of the impairment of functioning in all areas carried by ADHD, eventually bringing about secondary demoralization with associated decreased hedonic ability, sleep disorders and irritability [21].

Anxiety disorders are also very common in adults with ADHD, as documented in several studies that reported prevalence rates of anxiety disorders of around 50% [15,16,20]. Anxiety disorders have been associated with the hyperactive/impulsive clinical presentation in adults with ADHD, where a diagnosis of anxiety can be made in up to 60% of patients [20]. However, it should be noted that anxiety has been associated with reduction of impulsivity in children [22], therefore this aspect warrants further research.

Several studies have investigated the association between ADHD and personality disorders, finding rates of comorbidity ranging from 10 to 75% in adult ADHD samples [23,24,25,26,27,28]. Among personality disorders, the most prevalent ones are grouped in cluster B, namely borderline and antisocial disorders [29,30]. Symptoms such as impulsivity and emotional dysregulation, sensation seeking and comorbidity with substance abuse overlap in borderline personality disorder and ADHD [24,31]. The former should be hypothesized when patients display other characteristics that are not typical of ADHD, such as frantically avoiding real/imagined abandonment, suicidal and self-harm behaviors, chronic feelings of emptiness or severe stress-related dissociation. Clinical studies on adults with ADHD found comorbid borderline disorder in rates between 18 and 34% [29,32,33]. On the other hand, when looking at patients with borderline personality disorders, several studies found similarly high comorbidity rates of ADHD in 25–27% of cases [26,27,34].

Similarly, the diagnosis of ADHD put patients at risk for substance abuse and eventually the development of SUDs. Both subjects with ADHD and substance abuse are at increased risk for the other condition, although it is believed that ADHD more often predates the onset of SUDs, since illicit substances are often used as self-medication in the effort to deal with ADHD symptomatology [35]. The most commonly abused substances among patients with ADHD are nicotine, alcohol, cannabis and cocaine [36,37]. Clinical studies found even high prevalence rates of comorbid SUDs in adults with ADHD [38,39]. Dirks et al. found that a quarter of the patients surveyed with SUD met the diagnostic criteria for ADHD [38]. In a prospective study, adolescents with ADHD at baseline were 47% more likely at risk of developing SUDs in young adulthood [39]. Moreover, the presence of ADHD may significantly influence the course of SUDs, being associated with an early onset of substance use, higher rates of polyabuse, lower likelihood of achieving abstinence and hampering of treatment adherence [40,41]. Looking at the phenomenon the other way round, the International Collaboration on ADHD and Substance Abuse (ICASA), a network of 28 centers located in several countries founded with the aim of collecting data regarding ADHD and SUD, showed that one in six drug addicts also have comorbid ADHD. These patients reported greater exposure to childhood trauma, slowed infant development, issues in emotional control, risky behaviors and low educational attainment [42].

## 3. ADHD and Bipolar Disorder

Bipolar disorder (BD) is characterized by the alternation of mood episodes and variable periods of euthymia, which lead to worse overall functioning and quality of life. Several symptoms encountered in ADHD are common in BD, particularly during elated phases, making differential diagnosis often challenging. However, the existing differences in the phenomenology of the two disorders help guide the clinicians out of the maze. The core ADHD dimension of inattention is present during mood episodes; however, in ADHD it is frequently reported as a tendency to wander from one thought to another, whereas during hypomanic episodes it is often described as a peculiar clarity of thoughts, and during manic episodes it is the consequence of thought acceleration, leading to the typically observed distractibility. The impulsive dimension is also commonly found in BD during euphoric phases, although in those cases it can be often drawn back to inflated self-esteem or grandiosity, eventually leading to underestimating the consequence of actions. Similarly, the inability to stop and relax typical of ADHD can be observed in patients with BD during both anxious depression and manic episodes, although in the latter it usually takes the shape of increased goal-directed activity.

Some other clinical characteristics can help clinicians in differentiating BD from ADHD. First, some intraepisodic features typical of BD are not observed in ADHD: for instance, the increase in sexual activity often reported during manic phases is not reported in ADHD. Furthermore, sleep problems express differently: sleep deprivation in ADHD, whenever it occurs, is usually accompanied by discomfort and fatigue; on the other hand, during manic phases sleep need is reduced, oftentimes without any concomitant physical discomfort. Finally, psychotic symptoms, which can occur during severe mood episodes, are not observed in patients with ADHD, regardless of the severity of the disorder [43].

Course characteristics also help disentangling BD from ADHD. First and most importantly, BD has an oscillating course, with phases clearly different from one another. Therefore, in patients with BD, symptoms such as distractibility, impulsivity and hyperactivity fluctuate over time. On the contrary, ADHD symptomatology is usually stable over time [44]. Secondly, age of onset differs between the two disorders: usually during childhood for ADHD, with the first symptoms occurring before the age of 12, while the age of onset of BD peaks in late adolescence or early adulthood [45]. Commonalities and differences between ADHD and BD are presented in Table 1.

However, besides diagnostic overlapping, ADHD and BD often co-occur, as demonstrated by several studies. The National Comorbidity Survey-Replication (NCS-R), one of the broadest and most representative mental health survey conducted in the USA, estimated the prevalence of ADHD in 4.4% of 3199 respondents. Among those, comorbid BD could be diagnosed in the 21.2% [20]. In Europe, two large nationwide population studies conducted in Norway and Sweden investigated the prevalence of BD in respectively 40,000 and 61,000 subjects with a diagnosis of ADHD, finding quite homogeneous rates of BD in 8.9–9.4% of men and 13.5–18% of women [46,47]. Looking at the issue the other way round, in BD respondents from the NCS-R the comorbidity with ADHD was as high as 31.4%. Subjects with BD had a 6.7-times higher risk for co-occurring ADHD than the US general population [48]. In a further study from the WHO Mental Health Survey initiative conducted over 11 countries, lifetime ADHD could be diagnosed in the 19.8% of 1573 subjects with BD [49].

Clinical studies also reported high comorbid figures between ADHD and BD. In an early report on the first 1000 participants of the Systematic Treatment Enhancement Program for BD, the researchers found that comorbid ADHD could be diagnosed in 9.5% of BD patients, a figure that rose to 14.7% in males. Comorbid anxiety and SUDs, together with a greater burden of disease, were more frequent in patients with both conditions [50]. McIntyre and colleagues, in a subsequent report from the International Mood Disorders Collaborative Project, found that 17.6% of patients with BD interviewed with a structured diagnostic interview had comorbid ADHD. These patients had an earlier age at onset, more comorbid anxiety disorders and a lower quality of life [51]. In a Swiss study on 138 BD patients referred to a specialized mood disorders clinic, around half of them scored positive on the Adult ADHD Self-Report Scale. However, a correct diagnosis of ADHD was made in 20% of patients, suggesting that the overlapping symptomatology between ADHD and BD could lead to false positives when using a screening tool; therefore, a thorough diagnostic interview should always follow. Again, patients with comorbid ADHD had a younger age at onset and a higher risk of alcohol and substance abuse than those with BD only [52]. More recently, Pinna and colleagues investigated 703 patients with BD, finding that 255 of them (36%) had co-occurring ADHD. Patients with the two disorders had less successful school performance, less work and relational stability and a greater risk of substance abuse and suicide attempts [53].

Several longitudinal studies have tried to assess whether children with ADHD would develop (hypo)manic episodes over time. In the first, a 10-year follow-up study, boys with ADHD had a 7.9-times higher morbidity risk than healthy controls for developing BD by the age of 21 [54]. In a similar 11-year longitudinal study from the same research group, conducted on girls with ADHD and healthy controls, girls with baseline ADHD had an even higher rate than males, a 10-fold risk for developing BD by the age of 22 compared with controls [55]. Two more longitudinal studies investigated the likelihood of conversion to BD in adolescents with major depression with or without ADHD. In the first, Biederman and colleagues followed up on 168 adolescents with a history of major depression for 7 years: in those with comorbid ADHD, a manic episode occurred in 28% of cases, versus only 6% in those without ADHD [56]. Finally, a Taiwanese nationwide register study followed up on 58,000 young adults with major depression at baseline, of which around 1200 had a diagnosis of ADHD. After 10 years, the 19% of patients with baseline ADHD comorbidity would be diagnosed with BD versus the 11% of controls, with a 50% higher risk of developing BD after controlling for several confounding factors, among all psychiatric comorbidity [57].

Some studies assessed ADHD–BD comorbidity according to the type of BD. The above-mentioned population studies, the NCS-R and WHO collaborative study, reported very similar ADHD comorbidity rates among patients with BD type I and II [48,49]. Clinical studies retrieved mixed results, and a recently published meta-analysis found that comorbidity rates of ADHD did not differ in patients with BD type I or type II [58]. Unfortunately, neither population nor clinical studies have evaluated the clinical characteristics and prognostic implications of having a comorbid BD type I or II, therefore specific recommendations regarding type of BD cannot be drawn.

In conclusion, the reviewed clinical studies found that around 10–20% of adult patients with BD has comorbid ADHD, with even higher rates in those with earlier age at onset [59]. The abovementioned meta-analysis, pooling together population and clinical studies, found a combined prevalence of ADHD in the 17% of adult patients with BD [58]. On the other hand, youths with ADHD are more at risk to develop BD in young adulthood, with incidence rates of 7–21% [43,44]. The comorbidity between ADHD and BD appears to be a negative prognostic feature, since it may confer a greater severity and liability for other psychiatric disorders together with a worse overall functioning and burden of disease.

Bipolar disorder is a psychiatric condition particularly at risk for suicidal behaviors and committed suicide. On the other hand, ADHD can also increase suicide risk [60,61,62]. However, the effect of comorbidity between ADHD and BD on suicidality has not been comprehensively assessed to date. Lan and colleagues, in a cohort study, compared suicide rates in 500 adolescents with ADHD and BD versus 1500 age- and sex-matched adolescents with BD alone. At follow-up, patients with ADHD had committed suicide twice more often than those without, with a hazard ratio of 2.38 [63]. Another study, after controlling for demographic factors and psychiatric comorbidities including alcohol use disorders and substance use disorders, found that ADHD was still an independent risk factor for teenage suicide attempts [64]. The increased risk of suicidal behavior can be explained by the fact that patients with bipolar disorder and ADHD had a higher prevalence of disruptive behavior disorders and impulsivity, which has been associated with greater risk of attempted and completed suicide [30,63]. The recent observation of a reduction of suicide attempts and non-suicidal self-injury events in adult ADHD–BD patients six months after stimulant medication initiation [65] further strengthens this view. Moreover, the described greater severity of BD conferred by ADHD can further explain the increased suicide risk.

## 4. Treatment Implications of ADHD–BD Comorbidity

Since not stabilized BD can express with mood swings, impulsivity, hyperactivity and inattention, symptoms that overlap with ADHD presentation, the first goal of ADHD–BD comorbidity treatment should always be mood stabilization. Moreover, after being properly stabilized, some ADHD/BD patients might even not fulfill diagnostic criteria for ADHD anymore [66]. However, many patients complain of residual symptoms such as memory deficits and concentration difficulties, which are most likely due to the ADHD comorbidity [66]. In these cases it is advisable to add specific ADHD medications such as methylphenidate (MPH), atomoxetine or amphetamine salts, which have proved effective and well tolerated in both children and adults with ADHD. Nevertheless, only a few studies have addressed the efficacy and tolerability of these psychopharmacologic interventions in subjects with ADHD and comorbid BD. Specifically, a point of debate relates to the possible induction of manic episodes in patients exposed to ADHD medications such as stimulants or atomoxetine.

Early reports suggest a higher risk of treatment-emergent manic episodes in patients with ADHD treated with MPH, although more recent studies mitigated this association. In a retrospective study, DelBello and colleagues found an association between a past history of treatment with stimulants and an earlier onset of BD in a group of adolescents with ADHD, suggesting that stimulant exposure could accelerate the onset of BD [67]. In another study, 60 cases of stimulant-induced psychotic or manic symptoms were reviewed: in 92% of cases, symptoms were characterized by a brief duration, with recovery between 2 and 7 days of discontinuation or lowering MPH [68]. On the flip side, a study conducted on 289 children with ADHD treated with MPH showed no adverse outcomes of MPH treatment in those with manic symptoms at baseline; stimulant-treated children even showed a reduction in manic symptoms [69]. In more recent years, two studies have further contributed to clarify the matter. A Taiwanese large register study on 145,000 children newly diagnosed with ADHD and matched controls, followed up for up to 12 years, found that MPH-treated patients for more than one year had a reduced risk of new-onset BD compared to those not treated [70]. Furthermore, in a large Swedish registry study on 2307 adults with BD who later initiated MPH for concurrent ADHD, the authors found no evidence for a positive association between MPH and treatment-emergent mania among those on concomitant mood stabilizers. On the contrary, those treated with MPH alone had a 6.7-times increased rate of manic episodes within three months of medication initiation [71].

Regarding atomoxetine, in a first retrospective chart review on 7 pediatric patients with BD and ADHD already treated with mood stabilizers, no hypomania or mania was reported with the use of adjunctive atomoxetine over mood stabilizers for a period up to 18 months [72]. Similarly, in a subsequent small 8-week open-label study on 12 youth with ADHD and comorbid BD, atomoxetine in adjunct of a mood stabilizer did not induce manic or mixed episodes, although treatment was discontinued in two patients due to worsening of mood symptoms [73]. In an Italian pharmacovigilance study on 781 youths aged 6–18 years, atomoxetine caused a hypomanic episode in only one exposed patient [74]. Finally, in the abovementioned Taiwanese registry study, short-term treatment with atomoxetine in 145 children with ADHD did not correlate with a higher risk of BD onset [70]. Overall, akin to what occurred with MPH, these studies show a substantially low probability of treatment-emergent manic episodes when atomoxetine is associated with a mood stabilizing medication. However, five case reports described hypomanic or manic switches with atomoxetine. [75,76,77,78,79] Therefore, precautionarily, both the European summary of product characteristics and the US label state that treatment-emergent mania “without a prior history of psychotic illness or mania can be caused by atomoxetine at usual doses” [80].

Besides MPH and atomoxetine, amphetamine salts were studied in patients with ADHD–BD comorbidity. In a first study, youth patients with manic symptoms were initially treated with divalproex sodium for 8 weeks, showing a reduction of manic symptoms but no effect on ADHD symptomatology. At the end of this open-label phase, youths randomized to mixed amphetamine salts in adjunct to divalproex showed a significant reduction of ADHD symptoms, with only one patient experiencing a worsening in manic symptoms [81]. More recently, McIntyre and colleagues tested the effects of 4 weeks of adjunctive lisdexamfetamine in 40 adults with ADHD–BD comorbidity. Lisdexamfetamine was well tolerated and proved beneficial on ADHD and depressive symptoms, again without inducing any treatment-emergent hypomanic or manic episode [82]. Finally, alongside stimulants such as modafinil and armodafinil, lisdexamfetamine is the only stimulant marketed for use in ADHD to be studied as an add-on strategy for treatment-resistant bipolar depression. McElroy and colleagues randomized 25 adults with bipolar depression, which had been treated with mood stabilizers for 4 weeks, to lisdexamfetamine or placebo. After 8 weeks, patients on the adjunctive stimulant significantly improved compared to the placebo; the treatment, except for a single case of drug misuse, was overall well tolerated. None of the treated subjects experienced treatment-emergent manic or hypomanic symptoms [83].

## 5. Conclusions

Adult ADHD is often characterized by symptoms such as impulsivity, distractibility and restlessness, which clearly overlap with BD symptomatology, thus making differential diagnosis between the two disorders a challenge. Moreover, ADHD and BD often coexist, as highlighted by several population and clinical studies. Adults with ADHD and comorbid BD are a particularly critical group of patients showing a severe and burdensome clinical picture, with a lower quality of life, a higher number of mood episodes, an increased prevalence of substance abuse and dependence, and a worse overall functioning. In spite of that, only a few studies have investigated the best treatment modalities in these complex patients, therefore recommendations should still be considered as preliminary. In short words, when ADHD and BD co-occur, mood stabilization should be the first goal of treatment. When a mood stabilizer is in place, the augmentation with stimulant medications is effective in ameliorating the ADHD symptomatology. In these cases, the possibility of a treatment-emergent (hypo)manic switch should be taken into account, although emergence is uncommon when proper mood stabilization is in place. Finally, adults with ADHD and comorbid bipolar depression may benefit from the use of lisdexamfetamine.

## Figures and Tables

**Table 1:** Similarities and differences between ADHD and BD.

Characteristic	ADHD	BD
**Age at onset**	Childhood	Early adulthood
**Course**	Stable	Episodic
**Symptoms**	Labile, dysphoric mood Reduced self-esteem Distractibility perceived as thought wandering, without objective acceleration Restlessness, fidgetiness	Persistently euphoric, elevated or irritable mood Inflated self-esteem or grandiosity Distractibility due to acceleration of thought Increased goal-directed activity
**Sleep**	Usually not affected	Decreased need to sleep
**Sexuality**	Not affected	Increased (hypo- or mania)
**Psychosis**	Absent	Possible
